# Predictive factors for post-therapeutic biochemical discordance in acromegaly: a monocentric analysis of 156 cases

**DOI:** 10.1007/s11102-025-01547-8

**Published:** 2025-06-22

**Authors:** Dimitrios Emmanouilidis, Witold Polanski, Tareq Juratli, Stephan B. Sobottka, Elena Tsourdi, Matthias Gruber, Thomas Pinzer, Ilker Y. Eyüpoglu

**Affiliations:** 1 Department of Neurosurgery, Faculty of Medicine and University Hospital Carl Gustav Carus,, Technische Universität Dresden, Dresden, Germany; 2 Department of Medicine III, Technische Universität Dresden, Dresden, Germany

**Keywords:** Acromegaly, Biochemical discrepancy, Biochemical outcome, Predictive factors, Transsphenoidal surgery

## Abstract

**Purpose:**

Biochemical remission is the primary treatment goal in acromegaly. However, some patients experience biochemical discordance between growth hormone (GH) and insulin-like growth factor-I (IGF-I) levels following multimodal therapy, complicating disease assessment and management. This study aims to identify predictive factors associated with post-therapeutic biochemical discrepancy.

**Methods:**

We conducted a retrospective monocentric analysis of 156 patients with GH-producing pituitary adenomas (PAs) who underwent transsphenoidal surgery between 1984 and 2017. Biochemical outcomes were classified into four groups: group 1 (biochemical remission), group 2 (isolated GH normalization), group 3 (isolated IGF-I normalization), and group 4 (persistent acromegaly). Predictive factors for biochemical discrepancy were assessed, including demographic data, tumor characteristics, medication, irradiation, follow up duration, and disease recurrence.

**Results:**

The median age of the cohort was 48.2 years, with a female predominance (61.5%). Most PAs were macroadenomas (79.6%) and invasive (53.9%). Biochemical remission was achieved in 69.9%, while 19.2% exhibited biochemical discrepancy. Univariate analysis identified overall medication (pre- and/or postoperative), irradiation, and invasive PAs as significant factors associated with biochemical discordance. Logistic regression confirmed medication as the most influential predictor, with irradiation as a potential contributing factor. Disease recurrence was the only distinguishing factor between persistent acromegaly and biochemical discrepancy.

**Conclusion:**

Overall medication use is the strongest predictor of biochemical discrepancy, with irradiation potentially contributing. No clear distinguishing factors between biochemical discordance and persistent acromegaly were identified, except from disease recurrence. Managing patients with biochemical discrepancy similarly to those with persistent acromegaly may be advisable. Further research is needed to refine treatment strategies.

**Supplementary Information:**

The online version contains supplementary material available at 10.1007/s11102-025-01547-8.

## Introduction

Acromegaly is a chronic and rare endocrine disorder characterized by excess growth hormone (GH) secretion, resulting in elevated levels of insulin-like growth factor-I (IGF-I). In the vast majority of patients, the underlying cause is a benign somatotropic pituitary adenoma (PA) with autonomous GH hypersecretion. The disorder leads to progressive physical changes and a broad range of systemic comorbidities, which contribute to increased morbidity and mortality [1–5]. Timely diagnosis and intervention are crucial, as early intervention is associated with improved clinical outcomes and normalization of life expectancy [6–9]. However, even after therapy, not all comorbidities fully resolve [8, 10, 11]. Given the complexity of acromegaly, management is best conducted in multidisciplinary specialized centers [12–14].

Transsphenoidal surgery is the first-line treatment for PAs, followed by medical therapy, with irradiation being reserved for refractory or recurrent cases [3, 14–16]. The primary therapeutic objective is achieving biochemical remission. The 2010 acromegaly consensus established criteria for “controlled disease”, including age- and sex-matched normalization of IGF-I levels and random GH levels < 1 µg/L or nadir GH levels < 0.4 µg/L following an oral glucose tolerance test (OGTT) [5]. The most recent 2024 acromegaly consensus refined these definitions, reinforcing biochemical remission as the key therapeutic target, distinct from biochemical control. Due to the challenges in establishing universal GH thresholds– owing to multiple influencing factors - it was emphasized that age-adjusted IGF-I levels measured 12 weeks post-surgery serve as a primary marker of surgical success [12].

Achieving full biochemical remission remains challenging, even within specialized centers using well-calibrated GH and IGF-I assays. In some cases, biochemical discordance occurs, defined by persistently elevated levels of either GH or IGF-I levels, but not both [3, 5, 12]. This discordance complicates disease assessment and may lead to misinterpretations, potentially impacting prognosis and subsequent therapeutic decisions. Current guidelines offer no clear recommendations for the management of patients with biochemical discordance. These individuals may be at risk of disease recurrence or may continue to experience active acromegaly despite a discordant biochemical profile [17].

In this study, we aimed to identify predictive factors for biochemical discrepancy between GH and IGF-I levels following multimodal therapy. By conducting a retrospective analysis with long-term follow up and evaluating multiple associated factors, we sought to provide further insight into this complex and poorly understood patient group.

## Materials and methods

### Study design and patients

We conducted a comprehensive retrospective, monocentric analysis of patients with GH-secreting PAs who underwent surgery between 1984 and 2017. All surgical procedures were performed by the same neurosurgeon using a microsurgical transnasal transsphenoidal approach. The primary focus of our analysis was the biochemical outcome after multimodal therapy. Patients were classified based on the 2010 acromegaly consensus criteria into four distinct outcome groups:

- Group 1: Biochemical remission (normal GH and IGF-I levels).
- Group 2: Isolated GH normalization.
- Group 3: Isolated IGF-1 normalization.
- Group 4: Persistent acromegaly (elevated GH and IGF-I levels despite therapy).

To identify predictive factors for biochemical discrepancies, we analyzed the impact of various parameters on the long-term dynamic course of the disease, including demographic data, tumor-related characteristics, medication use, and irradiation.

The inclusion criteria were as follows: (a) PA as the cause of acromegaly, (b) clinically, biochemically, and histopathologically confirmed acromegaly, (c) microscopic transnasal transsphenoidal surgery, (d) first surgery performed at our hospital, and (e) availability of postoperative biochemical follow up data at least 12 weeks postoperatively. The initial cohort included 187 patients. Each patient was individually reviewed for compliance with the inclusion criteria using the hospital documentation system and outpatient records. A total of 31 patients were excluded for the following reasons: 23 patients lacked adequate postoperative biochemical follow up data (most often limited to immediate postoperative results), two patients had a suspected diagnosis of acromegaly that could not be confirmed, three patients had undergone primary surgery at an external center, one patient did not undergo surgical treatment, and two patients underwent transcranial surgery. Ultimately, our cohort consisted of 156 patients. The retrospective analysis of acromegaly patients was approved by the local Ethics Committee of TU (Technische Universität) Dresden (approval number: BO-EK-383082020).

### Statistical analysis

Data analysis was performed using SPSS (version 23.0, IBM, NY, USA). Descriptive statistics were applied to evaluate frequency distributions. Group comparisons were conducted using the Mann–Whitney U test for non-normally distributed continuous variables and the Kruskal-Wallis test for comparisons involving three or more groups. Categorical variables were analyzed using Fisher’s exact test or Pearson’s chi-squared test, with Bonferroni correction applied for multiple comparisons when necessary. Statistical significance was set at *p* < 0.05 (two-tailed), with *p* < 0.001 considered highly significant. All results were presented with 95% confidence intervals (CIs). Following univariate analysis, binary logistic regression analysis was performed, with odds ratios (ORs) calculated to quantify associations.

### GH and IGF-I levels

For statistical analysis, the highest recorded preoperative random GH value was used. Postoperatively, both the random GH value and the nadir GH value from the OGTT were considered. IGF-I levels were evaluated using the highest recorded value adjusted for age and sex. To account for patient aging and the resulting shifts in the IGF-I reference range, IGF-I levels were expressed as a percentage of the upper limit of normal (ULN) at each time point. Levels exceeding 100% were classified as pathologically elevated, while those at or below 100% were considered normal.

### GH and IGF-I assays

Serum GH levels were measured using radioimmunoassays (RIA) for patients before the 1990s. In the 1990s, the Abbott IMx analyzer, which combined a fluorescent polarization immunoassay module and a microparticle enzyme immunoassay system, was used until July 2005. From that point onward, chemiluminescent immunoassays (CLIA) were employed, starting with the Liaison assay until April 2008, followed by the Immulite 1000 analyzer until March 2014, the Liaison XL until November 2021, and the IDS-iSYS, which remains in use to the present.

IGF-I levels were measured using RIA until early 2006. From then on, CLIA were applied, initially with the Immulite 1000 until February 2013, followed by the Liaison XL until November 2021, and subsequently the IDS-iSYS, which is currently in use.

### Pituitary adenomas

PAs were classified based on their maximum diameter in sagittal magnetic resonance imaging (MRI) into microadenomas and macroadenomas, with macroadenomas defined as having a diameter of at least 10 mm. Adenoma size was measured in skull computed tomography (CT) scans before the MRI era [18]. Additionally, PAs were categorized according to their invasiveness into the cavernous sinus, based on the Knosp grading system. PAs were classified as non-invasive (Knosp grades 0–2) or invasive (Knosp grades 3–4) [19].

Recurrent and residual PAs were considered a single entity due to their similar biochemical consequences and were collectively referred to as “disease recurrence”.

Reoperation or radiation therapy was only performed if a PA was detectable on MRI.

## Results

### Study population and characteristics of pituitary adenoma

The final study population consisted of 156 patients, with a female predominance (61.5%) (Table 1). The median age at the first surgery was 48.2 years (range: 15–78.2 years) (Table 2). Among them, two patients were adolescents (15 and 16 years old). A total of 28 patients underwent surgery between 1984 and 1993, 41 between 1994 and 2003, 62 between 2004 and 2013, and 25 in the final three years from 2014 to 2017.

**Table 1:** Study population and characteristics of pituitary adenomas

		Patients (%)
**Gender** (*n* = 156)		
	Males	60 (38.5%)
	Females	96 (61.5%)
**Characteristics of PA**		
Size (*n* = 151)	Microadenoma	31 (20.4%)
	Macroadenoma	121 (79.6%)
Invasiveness (*n* = 141)	Invasive	76 (53.9%)
	Non-invasive	65 (46.1%)

PA pituitary adenoma

**Table 2:** Age of patients at first surgery

Age at first surgery	Median (range)	Mean (95% CI, SD)	*p*-value
Overall population (*n* = 156)	48.2 (15.0-78.2)	48.5 (95% CI: 46.3–50.7, SD: 14.1)	
Microadenoma (*n* = 31)	58.3 (25.2–73.8)	56.4 (95% CI: 51.8–61.0, SD: 12.5)	*p* < 0.001^a^
Macroadenoma (*n* = 121)	45.3 (15.0-78.2)	46.5 (95% CI: 43.9–49.0, SD: 14.0)	

CI confidence interval, SD standard deviation

a Mann–Whitney U test

Most PAs were macroadenomas (79.6%), and invasive PAs (53.9%) were slightly more frequent than non-invasive PAs. Patients with macroadenomas were significantly younger at the time of surgery (median age: 45.3 years, *p* < 0.001) (Table 2).

### Medication related data

Preoperative medication was administered to 69.2% of the patients, with 80.5% receiving somatostatin receptor ligands (SRLs). Postoperative medication was given to 28.8% of the patients, with 64.4% receiving SRLs, while pegvisomant was prescribed in 13.3% (6 patients) (Supplementary Table 1). The use of both preoperative and postoperative medication was independent of PA size and invasiveness (Table 4b).

### Biochemical outcome and biochemical discordance

Biochemical outcomes following multimodal therapy were classified into four distinct groups. Group 1 (69.9%) comprised patients who achieved biochemical remission, while group 2 (5.1%) and group 3 (14.1%) included patients with isolated normalization of GH or IGF-I, respectively. Group 4 (10.9%) represented persistent acromegaly, characterized by elevated GH and IGF-I levels.

Outcome groups 2 and 3 consisted of patients exhibiting biochemical discordance during long-term follow up assessments. Together, these groups accounted for 19.2% of the entire cohort and will henceforth be referred to as the “discrepancy group” (Table 3).

**Table 3:** Biochemical outcome groups of acromegaly patients after multimodal therapy

Outcome groups	GH	IGF-I	Patients, *n* = 156 (%)
Group 1	normal	normal	109 (69.9%)
Group 2	normal	elevated	8 (5.1%)
Group 3	elevated	normal	22 (14.1%)
Group 4	elevated	elevated	17 (10.9%)
Discrepancy group (combined group 2 and 3)	GH or IGF-I elevated, but not both		30 (19.2%)

GH growth hormone, IGF-I insulin-like growth factor I

### Univariate analysis of factors associated with biochemical outcome

We examined multiple factors in relation to biochemical outcomes, including gender, age at first surgery, preoperative random GH levels, preoperative IGF-I levels, size and invasiveness of PAs, preoperative medication, postoperative medication, overall medication (combined preoperative and/or postoperative), irradiation, disease recurrence, and follow up duration. To evaluate the association of these factors with biochemical outcomes, particularly biochemical discordance, comparisons were conducted in three ways: (a) across all four outcome groups (1 to 4), (b) between group 1, the discrepancy group, and group 4, and (c) between group 1 and the discrepancy group.

### Gender and age at first surgery

Although females had a higher remission rate (69.8%) and males exhibited a higher discrepancy rate (46.6%), no statistically significant differences were observed regarding gender. Similarly, age at the time of the first surgery showed no significant differences across groups, although patients with persistent acromegaly (outcome group 4) were the youngest (median: 39.7 years) (Table 4a).

**Table 4a:** Univariate analysis of factors associated with biochemical outcome

Outcome groups	Gender, male (%) ^a^ female (%) ^b^		Age at first surgery, median in years (95% CI)	Preoperative GH, median in µg/L (95% CI)	Preoperative IGF-I, median in % of the ULN (95% CI)	Size of PA ^c^, macroadenoma (%) ^d^ microadenoma (%) ^e^		Invasiveness of PA ^f^, invasive (%) ^g^ non-invasive (%) ^h^	
Group 1	42 (38.5%)	67 (69.8%)	50.5 (95% CI: 46.8–52.1)	11.2 (95% CI: 15.8–25.6)	286 (95% CI: 263.9–318.5)	83 (68.6%)	24 (77.4%)	49 (64.5%)	50 (76.9%)
Group 2	6 (75%)	2 (2.1%)	54.1 (95% CI: 42.5–62.7)	6.9 (95% CI: 3.3–10.9)	386.1 (95% CI: 323.2–443.3)	7 (5.8%)	1 (3.2%)	6 (7.9%)	2 (3.1%)
Group 3	8 (36.4%)	14 (14.6%)	42.7 (95% CI: 40.0–52.6)	21.8 (95% CI: 14.9–43.5)	266.5 (95% CI: 219.5–339.0)	17 (14%)	4 (12.9%)	14 (18.4%)	6 (9.2%)
Group 4	4 (23.5%)	13 (13.5%)	39.7 (95% CI: 35.1–51.1)	25.4 (95% CI: 19.4–46.5)	279.8 (95% CI: 201.2–405.0)	14 (11.6%)	2 (6.4%)	7 (9.2%)	7 (10.8%)
Discrepancy group	14 (46.6%)	16 (16.7%)	45.8 (95% CI: 42.9–53.2)	13.9 (95% CI: 12.4–34.3)	298.5 (95% CI: 260.3–356.4)	24 (19.8%)	5 (16.1%)	20 (26.3%)	8 (12.3%)
**Comparisons**	**p-value**								
Groups 1 / 2 / 3 / 4	0.104 ^i^		0.287 ^k^	**0.008** ^k, m^	0.123 ^k^	0.749 ^i^		0.223 ^i^	
Group 1 / discrepancy group / group 4	0.293 ^i^		0.275 ^k^	0.062 ^k^	0.733 ^k^	0.587 ^i^		0.115 ^i^	
Group 1 / discrepancy group	0.332 ^i^		0.623 ^l^	0.612 ^l^	0.439 ^l^	0.545 ^i^		**0.04** ^i^	

a It refers to 60 male patients

b It refers to 96 female patients

c PA size is known for 152 patients

d It refers to 121 patients with macroadenomas

e It refers to 31 patients with microadenomas

f The invasiveness of the PA is known for 141 patients

g It refers to 76 patients with invasive PAs

h It refers to 65 patients with non-invasive PAs

i Pearson’s chi-squared test

k Kruskal-Wallis test

l Mann–Whitney U test

m Bonferroni correction was significant (*p* = 0.003) for outcome groups 2 and 4

**Table 4b:** Univariate analysis of factors associated with biochemical outcome

Outcome groups	Preoperative medication (%)	Postoperative medication (%)	Overall medication (%) ^e^	Irradiation (%)	Disease recurrence (%)	Follow up, median in months (95% CI)
Group 1	73 (66.9%)	24 (22%)	77 (70.6%)	6 (5.5%)	15 (13.7%)	69.0 (95% CI: 77.4–107.9)
Group 2	6 (75%)	1 (12.5%)	6 (75%)	0 (0%)	0 (0%)	47.7 (95% CI: 7.6–95.1)
Group 3	17 (72.3%)	13 (59.1%)	21 (95.5%)	5 (22.7%)	4 (18%)	95.5 (95% CI: 75.3-146.1)
Group 4	12 (70.6%)	7 (41.2%)	13 (76.5%)	3 (17.7%)	7 (41.2%)	120 (95% CI: 89.8.143.5)
Discrepancy group	23 (76.6%)	14 (46.6%)	27 (90%)	5 (16.7%)	4 (13.3%)	86.5 (95% CI: 66-123.7)
Overall population	108 (69.2%)	45 (28.8%)	117 (75%)	14 (8.9%)	26 (16.6%)	79.5 (95% CI: 83.5-107.9)
**Comparisons**	**p-value**					
Groups 1 / 2 / 3 / 4	0.785 ^a^	**0.002** ^a^	0.110 ^a^	**0.029** ^a^	**0.022** ^a^	**0.016** ^b^
Group 1 / discrepancy group / group 4	0.590 ^a^	**0.015** ^a^	0.094 ^a^	0.069 ^a^	**0.016** ^a^	0.080 ^b^
Group 1 / discrepancy group	0.309 ^a^	**0.007** ^a^	**0.033** ^d^	**0.045** ^a^	1.000 ^d^	0.671 ^c^

a Pearson’s chi-squared test

b Kruskal-Wallis test

c Mann–Whitney U test

d Fisher’s exact test

e Ιt indicates medication use preoperatively and/or postoperatively

### Preoperative GH and IGF-I levels

The highest preoperative GH values were observed in outcome group 3 (median: 21.8 µg/L) and outcome group 4 (median: 25.4 µg/L). A statistically significant difference was found across all outcome groups (*p* = 0.008), which remained significant (*p* = 0.003) after applying the Bonferroni correction for outcome groups 2 (median: 6.9 µg/L) and 4.

The highest preoperative IGF-I levels were recorded in outcome group 2 (median: 386.1%) and the discrepancy group (median: 298.5%). However, comparisons among the outcome groups did not reach statistical significance (Table 4a).

### Size and invasiveness of pituitary adenomas

Although macroadenomas exhibited a lower biochemical remission rate (68.6%) and a higher biochemical discordance rate (19.8%) compared to microadenomas (77.4% and 16.1%, respectively), adenoma size did not significantly influence biochemical outcomes (Table 4a).

Regarding invasiveness, invasive PAs were associated with a lower remission rate (64.5%) and higher biochemical discordance (26.3%) compared to non-invasive PAs (76.9% and 12.3%, respectively). Statistical significance (*p* = 0.04) was reached only in the comparison between outcome group 1 and the discrepancy group (Table 4a).

### Medication

No statistically significant differences were observed in preoperative medication use among the outcome groups.

In contrast, the administration of postoperative medication showed statistically significant differences across all group comparisons. The highest rate of postoperative medication was observed in outcome group 3, with 13 of 22 patients (59.1%), including four patients who received pegvisomant, a medication that inevitably increases GH levels (Supplementary Table 1).

Overall medication use, defined as administration either preoperatively and/or postoperatively, was recorded in 75.0% of the patients. The comparison between outcome group 1 (70.6%) and the discrepancy group (90%) reached statistical significance (*p* = 0.03) (Table 4b).

### Irradiation

Radiation therapy, either conventional or stereotactic, was administered to 8.9% of the patients. The highest rate was observed in the outcome group 3 (22.7%). The comparison across all four outcome groups was statistically significant (*p* = 0.029). Additionally, a direct comparison between group 1 and the discrepancy group also showed statistical significance (*p* = 0.045) (Table 4b).

### Disease recurrence

Disease recurrence was observed in 16.6% of the patients, with the highest rate occurring as expected in outcome group 4 (41.2%). The comparison across all four outcome groups was statistically significant (*p* = 0.02). Also, the comparison between group 1, discrepancy group und group 4 was significant (Table 4b).

### Follow up

The median follow up duration was 79.5 months (range: 4–419 months; mean: 95.7 months, 95% CI: 83.4–107.9; SD: 77.2). A statistically significant difference was observed across biochemical outcome groups 1 to 4 (*p* = 0.016). After Bonferroni correction, significance was specifically attributed to the comparison between groups 2 (median: 47.7 months) and 4 (median: 120 months) (Table 4b).

### Logistic regression analysis of predictive factors for biochemical discrepancy

To identify predictive factors for biochemical discrepancy after multimodal therapy, we performed a binary logistic regression analysis, comparing the discrepancy group with both outcome group 1 (biochemical remission) and outcome group 4 (persistent acromegaly).

Initially, we conducted a regression analysis that included clinically relevant variables such as gender, age at first surgery, PA size and invasiveness, preoperative GH and IGF-I levels, preoperative medication, overall medication (combined pre- and postoperative), irradiation, and disease recurrence.

In the comparison between outcome group 1 and the discrepancy group, overall medication (*p* = 0.041) and irradiation (*p* = 0.043) were identified as significant factors. Conversely, when comparing outcome group 4 and the discrepancy group, none of the variables reached statistical significance (Table 5).

**Table 5:** Logistic regression analysis

Variables	Outcome group 1 - Discrepancy group			Outcome group 4 - Discrepancy group			
	*p*-value	OR	95%CI	Variables	*p*-value	OR	95%CI
Gender	0.940			Gender	0.297		
Age at first surgery	0.946			Age at first surgery	0.053		
Size of PA	0.694			Size of PA	0.728		
Invasiveness of PA	0.082			Invasiveness of PA	0.263		
Preoperative GH	0.427			Preoperative GH	0.231		
Preoperative IGF-1	0.558			Preoperative IGF-1	0.908		
Preoperative medication	0.070			Preoperative medication	0.681		
Overall medication	**0.041**	4.988	1.067–23.315	Overall medication	0.482		
Irradiation	**0.043**	4.957	1.055–23.299	Irradiation	0.961		
Disease recurrence	0.684			Disease recurrence	0.058		
**Reduced regression model** ^a^							
**Variables**	**p-value**	**OR**	**95%CI**	**Variables**	0.205		
Invasiveness of PA	0.058			Invasiveness of PA	0.305		
Overall medication	**0.040**	3.740	1.059–13.213	Overall medication	0.211		
Irradiation	0.058			Irradiation	0.932		
				Disease recurrence	**0.038**	4.550	1.090-18.987

OR odds ratio, CI confidence interval

a In the reduced regression model, only variables that were significant in the univariate analysis were included

To refine our model, we performed a reduced logistic regression, including only a subset of the variables mentioned above. We selected factors that were significant in the univariate analysis for outcome group 1 and the discrepancy group, namely PA invasiveness, overall medication, and irradiation (Table 4a and 4b). This analysis showed that only pre- and/or postoperative medication (overall medication) remained statistically significant (*p* = 0.04; OR: 3.740; 95% CI: 1.059–13.213) (Table 5).

For the comparison between outcome group 4 and the discrepancy group, the reduced model included the same variables, given their clinical relevance, along with disease recurrence, which was significant in the univariate analysis when outcome group 4 was considered (Table 4b). Here, disease recurrence emerged as the only significant predictor (*p* = 0.038; OR: 4.550; 95% CI: 1.090–18.987) (Table 5).

## Discussion

This study aimed to identify predictive factors for biochemical discrepancy following multimodal therapy in acromegaly. Unlike conventional classification that distinguish only between remission and persistent acromegaly, we introduced a four-group outcome classification to better characterize biochemical response patterns.

The discrepancy group, consisting of patients with isolated GH or IGF-I normalization, accounted for 19.2% of the cohort, emphasizing the clinical relevance of this subgroup. Given that patients with biochemical discordance do not meet full remission criteria yet differ from those with persistent acromegaly, an improved understanding for their prognosis and management is essential. Managing this patient group is crucial due to the potential risk of disease recurrence. Puder et al. described a trend toward increased mortality in biochemically discordant patients [20]. Studies published before 2014 have reported biochemical discordance rates ranging from 5.4 to 39.5%, with most cases exhibiting normal GH and elevated IGF-I levels [21]. In contrast, our cohort had a higher proportion of outcome group 3 patients with normal IGF-I levels. However, four patients in group 3 received pegvisomant, which inevitably increases serum GH levels due to its pharmacokinetics. Adjusting for this effect, the rate of outcome group 1 increases to 72.4% (*n* = 113), while the rate of outcome group 3 decreases to 11.5% (*n* = 18). Consequently, the discrepancy group (combined groups 2 and 3) accounts for a lower rate of 16.6% (*n* = 26).

It is also noteworthy that, excluding the four patients treated with pegvisomant, 44% (*n* = 8/18) of the remaining patients in group 3 received postoperative SRLs. This small subgroup, characterized by normalized IGF-I levels and elevated GH, may be consistent with the concept of extra-hepatic acromegaly proposed by Sebastian Neggers’ group [22]. Further research is warranted to better understand the clinical relevance and pathophysiology of this phenomenon.

Several factors have been identified as potential contributors to biochemical discordance, including gender, age, radiotherapy, and medical therapy, among others [3, 18].

The univariate analysis of our study revealed that invasive PAs, overall medication (pre- and/or postoperative), and irradiation were significantly associated with biochemical discrepancy when compared to outcome group 1 (biochemical remission). In each case, the rate of biochemical discordance was higher, suggesting that these factors may contribute to biochemical discrepancy (Tables 4a and 4b).

Regarding preoperative medication, the literature remains contradictory, and no clear recommendation exists for the routine use of SRLs in the preoperative setting [11, 13]. In our cohort, the administration of preoperative medication was guided by our intention to exhaust all available options to achieve postoperative biochemical remission. In particular, preoperative SRL therapy was aimed at improving tumor-associated conditions to facilitate complete adenoma resection. The high rate of preoperative medical treatment in our cohort (69.2%), predominantly with SRLs (80.5%), can be attributed in part to the relatively high proportion of macroadenomas (79.6%) and invasive pituitary adenomas (53.9%) in our patient group.

Postoperative adjuvant medical therapy was initiated in all cases solely based on the absence of biochemical remission in postoperative endocrine evaluations.

Additionally, preoperative GH levels were statistically significant in differentiating outcome group 2 (isolated GH normalization) from outcome group 4 (persistent acromegaly), with group 2 patients having the lowest preoperative GH levels overall. This aligns with their post-therapeutic biochemical profile, in which only GH normalizes. However, this pattern was not observed for preoperative IGF-I levels in outcome group 3, suggesting a different underlying mechanism (Table 4a). We hypothesize that this may be due to the limited number of GH receptors in peripheral tissues, affecting IGF-I synthesis.

Furthermore, the comparison between outcome groups 2 and 4 in relation to follow up duration was statistically significant, with group 2 patients (elevated IGF-I levels) having the shortest follow-up (Table 4b). This raises the hypothesis that IGF-I levels in these patients may normalize over time with extended follow up or, conversely, that the disease may progress, leading to an increase in GH levels as well.

Postoperative medication was also statistically significant across all comparisons in the univariate analysis, with outcome group 4 exhibiting the highest rate of postoperative medication use (Table 4b). This finding aligns with the expected clinical management of persistent acromegaly, reinforcing the appropriate and targeted use of postoperative medication in our cohort.

Finally, disease recurrence was statistically significant when comparing all four outcome groups, as well as in the comparison between outcome group 1, the discrepancy group, and outcome group 4. As expected, outcome group 4 exhibited the highest rate of disease recurrence in each comparison (Table 4b).

In this context, we would like to clarify once again that disease recurrence refers to both recurrent PAs, which lead to pathological GH and IGF-I levels indicative of persistent acromegaly, and residual PAs that could not be completely resected and therefore require further treatment, such as postoperative medical therapy or radiotherapy.

To gain a deeper understanding of the impact of predictive factors on biochemical discordance, we conducted a binary logistic regression analysis, comparing the discrepancy group with both outcome group 1 (biochemical remission) and outcome group 4 (persistent acromegaly).

Initially, we analyzed clinically relevant factors, including gender, age at first surgery, PA size and invasiveness, preoperative GH and IGF-I levels, preoperative medication, overall medication, irradiation, and disease recurrence. Postoperative medication was excluded, as its use was largely influenced by the biochemical outcome itself. Similarly, follow up duration was omitted, as patients with longer follow up were typically those who had not achieved biochemical remission.

The regression analysis comparing the discrepancy group and outcome group 1 identified overall medication and irradiation as significant predictive factors. However, in the reduced regression model, only overall medication remained significant, distinguishing biochemical discrepancy from remission (Table 5). It is important to note that special caution is required when interpreting patients receiving pegvisomant, as its pharmacokinetic effects influence GH levels, potentially impacting the classification of biochemical outcomes.

In contrast, the regression analysis comparing the discrepancy group and outcome group 4 did not reveal any significant factors. Only the reduced model demonstrated a significant role of disease recurrence, which is inherently associated with persistent acromegaly (Table 5). Consequently, no relevant predictive factors emerged to differentiate biochemical discrepancy from persistent acromegaly.

Given these findings, it may be advisable to manage patients with biochemical discordance similarly to those with persistent acromegaly, considering that biochemical remission remains the strongest predictor of clinical outcome [23].

### Limitations

The study has several limitations, the most significant being its retrospective nature.

A key limitation is the use of different immunoassays for GH and IGF-I over the long follow up, which may have affected the biochemical consistency of the cohort. Nevertheless, despite the variability in assays, a meaningful assessment of patients’ biochemical course and response to therapy was still possible. While the use of standardized, modern assays would have been ideal, this was not feasible due to the extended duration of the study. To enhance comparability, all patients were classified according to the 2010 acromegaly consensus criteria, regardless of when the biochemical tests were performed.

Another limitation concerns the diagnostic utility of random GH measurements. According to the most recent 2024 consensus on criteria for the diagnosis and remission of acromegaly, random GH plays a limited role in both diagnosis and assessment of therapeutic efficacy [12]. However, in our study we relied on the 2010 consensus criteria as the reference standard, which included random GH values as part of the diagnostic criteria [5].

Additionally, our study did not evaluate the histopathological characteristics of the PAs. Although all patients had a confirmed histopathological diagnosis of GH-secreting PA, many reports, especially those from earlier decades, lacked sufficient detail for meaningful analysis of histological subtypes.

Finally, the study did not analyze the specific role and effect of each pharmacological class on biochemical discordance. Further investigations, ideally large prospective multicenter studies, are necessary to draw more definitive conclusions in this regard.

## Conclusion

Biochemical discrepancy following multimodal therapy in acromegaly affects a significant proportion of patients. Our monocentric study aimed to identify predictive factors associated with biochemical discordance.

Our findings indicate that pre- and/or postoperative medication (overall medication) is the strongest predictor of biochemical discrepancy when compared to biochemical remission, with irradiation identified as a potential contributing factor. However, no predictive factors differentiated biochemical discrepancy from persistent acromegaly, except for disease recurrence, which is inherently linked to persistent disease. These findings underscore the clinical relevance of biochemical discordance, as it may indicate an ongoing pathological process. Given the absence of distinguishing predictors between biochemical discrepancy and persistent acromegaly, a similar management approach to persistent disease may be advisable.

Future studies with larger cohorts and extended follow up periods are essential to further clarify the natural history of biochemical discrepancy and establish evidence-based management strategies for these patients.

## Electronic supplementary material

Below is the link to the electronic supplementary material.

Supplementary Material 1

## Data Availability

The data that support the findings of this study are not openly available due to reasons of sensitivity and are available from the corresponding author upon reasonable request.
