# Supplementary material for: Predictive factors for post-therapeutic biochemical discordance in acromegaly: a monocentric analysis of 156 cases
Source: Pituitary. 2025 Jun 22;28(4):74. doi: 10.1007/s11102-025-01547-8 (PMC12183128; doi:10.1007/s11102-025-01547-8)
Supplement: Supplementary file 1 — Supplementary Material 1 [file 11102_2025_1547_MOESM1_ESM.docx]

| **Medication** | **Preoperative medication, n (%)** 108 (69.2%) ^a^ | **p-value** ^h^ | **Postoperative medication, n (%)** 45 **(**28.8%) ^a^ | **p-value** ^h^ |
| --- | --- | --- | --- | --- |
| SRLs | 87 (80.5%) ^b^ |  | 29 (64.4%) ^c^ |  |
| DA | 3 (2.8%) ^b^ |  | 4 (8.9%) ^c^ |  |
| SRLs and DA | 14 (12.9%) ^b^ |  | 2 (4.4%) ^c^ |  |
| Pegvisomant | 4 (3.7%) ^b^ |  | 6 (13.3%) ^c^ |  |
| SRLs and Pegvisomant | 1 (0.9%) ^b^ |  | 4 (8.9%) ^c^ |  |
|  | | | |  |
| **Microadenoma** ^d^ | 22 (71%) | 0.991 | 5 (16.1%) | 0.092 |
| **Macroadenoma** ^e^ | 86 (71.1%) |  | 38 (31.4%) |  |
|  | | | |  |
| **Invasive** ^f^ | 57 (75%) | 0.717 | 24 (31.6%) | 0.180 |
| **Non-invasive** ^g^ | 47 (72.3%) |  | 14 (21.5%) |  |

**Supplementary material**

**Supplementary Table 1** Medication related data

SRLs somatostatin receptor ligands, DA dopamine agonists

a It refers to overall population (n=156)

b It refers to 108 patients who received preoperative medication

c It refers to 45 patients who received postoperative medication

d It refers to 31 patients with microadenoma

e It refers to 121 patients with macroadenoma

f It refers to 76 patients with invasive PA

g It refers to 65 patients with non-invasive PA

h Pearson’s chi-squared test
